# The Use of Amiloride and Sodium-Glucose Cotransporter 2 Inhibitors in Cisplatin-Induced Hypomagnesemia: A Case Report and Review of the Literature

**DOI:** 10.7759/cureus.62546

**Published:** 2024-06-17

**Authors:** Caio T Heleno, Helena Miranda, Nico Gotera, Goetz Kloecker

**Affiliations:** 1 Hematology and Oncology, University of Louisville, Louisville, USA; 2 Medicine, Florida Atlantic University Charles E. Schmidt College of Medicine, Boca Raton, USA; 3 Clinical Internal Medicine, University of Texas Health San Antonio, San Antonio, USA

**Keywords:** nephrotoxicity, amiloride, sglt-2 inhibitor, cisplatin, hypomagnesemia

## Abstract

Cisplatin, a chemotherapy agent widely used since its FDA approval in 1978 for testicular cancer, is associated with nephrotoxicity and hypomagnesemia. Magnesium supplementation is not only a treatment for hypomagnesemia but also a well-established agent in preventing cisplatin-induced nephrotoxicity (CIN). Considering the challenges associated with intravenous magnesium use and even with the supplementation of oral forms, there is a need for drugs that effectively reduce urinary magnesium excretion. Amiloride and sodium-glucose cotransporter 2 inhibitors (SGLT2 inhibitors) have emerged as potential candidates. Amiloride is a well-known potassium-sparing diuretic that also has a hypomagnesemia effect seen in preclinical data. SGLT2 inhibitors are a drug class initially used in diabetes that was also observed to have positive effects on cardiovascular mortality, diabetic kidney disease, and hypomagnesemia. SGLT2 inhibitors were found to reduce hypomagnesemia in a meta-analysis study of 18 trials. However, these trials were not specifically designed for the evaluation of hypomagnesemia, and their current use in hypomagnesemia is considered off-label.

## Introduction

Cisplatin use is commonly associated with cisplatin-induced nephrotoxicity (CIN), with up to 25-33% of patients experiencing this adverse effect after a single dose of 50-75 mg/m^2^ [1,2]. Among those affected, hypomagnesemia is prevalent in approximately 90% of cases [3,4]. Because of the challenges of oral magnesium supplementation, especially diarrhea, alternative approaches are necessary. Here, we present a case of cholangiocarcinoma treated with cisplatin, gemcitabine, and durvalumab for a metastatic disease. The patient presented severe hypokalemia and hypomagnesemia secondary to cisplatin use. The hypomagnesemia was initially treated with oral magnesium and subsequently with amiloride and dapagliflozin with an improvement of the magnesium levels. This essay will review the current knowledge on amiloride use in hypomagnesemia and the role of sodium-glucose cotransporter 2 inhibitors (SGLT2) inhibitors. SGLT2 inhibitors were initially approved for diabetes use; however, more recently, they were seen to affect magnesium homeostasis. Large studies showed a reduction in urinary magnesium wasting, making SGLT2 inhibitors a promising class for hypomagnesemia treatment. Here, we will review the most important studies that support the use of SGLT2 inhibitors in hypomagnesemia treatment.

## Case presentation

**Figure 1:**
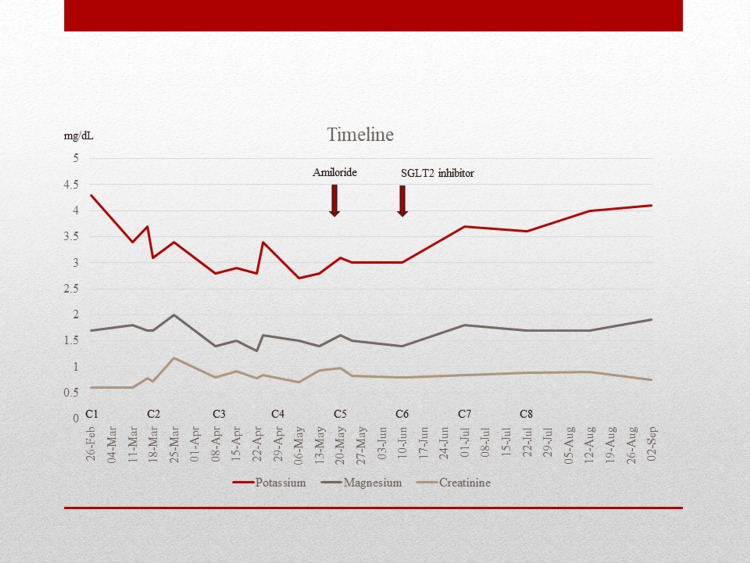
Timeline showing the time of the introduction of amiloride and sodium-glucose cotransporter 2 (SGLT2) inhibitors. Graphic showing potassium, magnesium, and creatinine levels according to dates and cycles of chemotherapy. The timeline goes from cycle one to eight and after chemotherapy.

A 57-year-old female, with a history of obesity, hypertension, and type 2 diabetes, was diagnosed with extrahepatic cholangiocarcinoma in August 2022 after cytology revealed adenocarcinoma. A month later, the patient underwent a duodenal-pancreatectomy, which revealed a final pathologic stage IIIA, pT3N2M0. The pathology report showed negative margins, with five out of 24 lymph nodes affected by adenocarcinoma. Because of these findings, the patient was referred for adjuvant chemoradiotherapy. The patient received radiation at a dose of 4,500 cGy/25 sessions with concurrent use of capecitabine. In March 2023, the patient had recurrent disease and was started using cisplatin 25 mg/m^2^ D1+D8, gemcitabine 1,000 mg/m^2^ D1+D8, and durvalumab 1,500 mg D1. The cycles were repeated every 21 days. After two cycles of the chemotherapy regimen, the patient presented with potassium of 2.8 mg/dL (normal range: > 3.5 mg/dL), magnesium of 1.4 mg/dL (normal range: > 1.7 mg/dL), and creatinine (Cr) of 0.60 mg/dL (normal range: < 1.1 mg/dL). The patient had symptoms of fatigue, weakness, nausea, and poor appetite that were related to chemotherapy treatment. The patient received potassium and magnesium intravenously and had a home prescription for oral potassium and magnesium. She began using magnesium oxide 400 mg/day, which had to be discontinued soon because of severe diarrhea. In the next cycles, the potassium levels were between 3.1 and 3.4 mg/dL using 40 mEq of potassium chloride extended-release daily. The magnesium levels were between 1.2 and 1.4 mg/dL (normal range: > 1.7 mg/dL), and Cr levels were between 0.60 and 0.90 during this period. In the fifth cycle of chemotherapy, the patient continued to present with persistent hypomagnesemia besides the use of 4g of intravenous magnesium on each chemotherapy day (D1+D8). At this moment, she started the use of amiloride 10 mg/day, and the potassium chloride was discontinued. In the first evaluation, after starting amiloride, the potassium levels were 3.7 mg/dL and magnesium was 1.4 mg/dL. The case was discussed with the diabetes team, who decided to change her diabetes regimen because of an A1C level of 9.5%, initiating the use of dapagliflozin 10 mg/day. She was already on the use of metformin. Nearly three weeks after starting dapagliflozin, the potassium levels were 3.9 mg/dL and magnesium levels were 1.8 mg/dL. She maintained normal range magnesium levels in the next evaluations, between 1.7 and 1.9 mg/dL. After eight cycles, the chemotherapy regimen was stopped, and she continued using durvalumab monotherapy. The patient continued using dapagliflozin even after the chemotherapy regimen was stopped. Two months after finishing the chemotherapy treatment, the use of amiloride was discontinued (Figure 1).

## Discussion

CIN commonly manifests as acute kidney injury (AKI), with a slow rise in serum creatinine observed after five to seven days of therapy. AKI can affect 25-33% of patients after a single dose of 50-75 mg/m^2^. However, it is important to note that the definition of nephrotoxicity can vary between studies, leading to discrepancies in epidemiological assessments and interpretations of the prevalence and severity of nephrotoxicity associated with cisplatin treatment [1,2]. CIN can occur earlier in patients with comorbid risk factors [2]. Cisplatin typically affects the tubulointerstitial structures of the kidney, while relatively sparing of the glomeruli. Therefore, glomerular filtration rate and creatinine level may not be the best indicators of CIN. Conversely, among those with CIN, the prevalence of hypomagnesemia is nearly 90% [3]. Electrolyte imbalances such as hypomagnesemia, hypocalcemia, and hypokalemia are early indicators of renal damage and can manifest before the onset of CIN [4-6].

Magnesium wasting is a common electrolyte abnormality in cisplatin-treated individuals. Glomerular filtration rate (GFR) and serum magnesium levels were significantly decreased following cisplatin administration at doses ≥50 mg/m^2^, in contrast to patients receiving cisplatin at a lower daily dose of 20 mg/m^2^. The lower daily dose regimen was observed to preserve GFR, but resulted in low magnesium levels, highlighting the importance of high peak plasma platinum concentrations for the toxicity related to the drug [6-8]. Hypomagnesemia also exhibits an increasing incidence with cumulative cisplatin dose, ranging from 41% after a single course to 100% of patients receiving six cycles of therapy [6]. Urinary magnesium wasting may persist even after discontinuation of cisplatin therapy and has been reported for more than six years following treatment cessation [3,9]. Drug-induced Gitelman syndrome has also been reported with cisplatin, and it can be permanent [9].

Kidney dysfunction following cisplatin administration is related to the exposure of tubular cells to cisplatin, which activates complex signaling pathways resulting in tubular cell injury and cell death. Risk factors associated with CIN include doses of cisplatin over 50 mg/m^2^, high peak plasma-free platinum concentrations, previous exposure to cisplatin, preexisting kidney damage, and concomitant use of other nephrotoxic agents. Additional risk factors include older age, hypoalbuminemia, female sex, smoking, a history of high blood pressure, and concomitant use of ifosfamide and paclitaxel. The higher toxicity related to women is explained in part by lower unbound cisplatin clearance in females [4]. In addition to its direct clinical manifestations, hypomagnesemia may exacerbate cisplatin toxicity [10].

The treatment of hypomagnesemia generally consists of intravenous or oral magnesium supplementation. The usage of high doses of magnesium may raise the plasma magnesium concentration, increasing the degree of urinary magnesium waste. When an intravenous magnesium infusion is given, an abrupt and temporary elevation in the plasma magnesium concentration will partially reduce the stimulus to magnesium reabsorption in the loop of Henle. Oral repletion, especially with sustained-release preparations, has the advantage of slow absorption, minimizing renal excretion of the administered magnesium while permitting the use of lower doses. The major limitation of oral magnesium supplementation is diarrhea. The use of sustained-release and lower doses minimizes this side effect [11]. Our case presented cisplatin-induced hypomagnesemia despite the use of a lower dose of 25 mg/day. The use of intravenous magnesium did not show long-lasting effectiveness, and the oral use of magnesium was discouraged because of diarrhea.

Cisplatin and magnesium

The use of magnesium has been associated with the prevention of CIN in patients with cisplatin-containing regimens. This toxicity reduction was noticed even with no difference in the degree of Δ serum magnesium level (lowest serum magnesium level minus baseline serum magnesium level) between the two groups [10-14]. A systematic review published in 2017 found four studies that specifically evaluated the role of magnesium supplementation in preventing CIN. The largest study retrospectively reviewed 496 thoracic malignancy patients treated with cisplatin (≥60 mg/m^2^)-containing regimens as first-time chemotherapy. Intravenous magnesium was preloaded with a dose of 8 mEq (1g) before each cycle. Multivariate analysis indicated that magnesium preload significantly reduced CIN with an odds ratio of 0.234 (95% confidence interval: 0.129-0.414), resulting in a reduction of 3.8-fold for the first cycle (p<0.001) and by 4.3-fold for all cycles (p<0.001) [12]. The study did not validate the association between CIN and the serum or urinary Mg level. The trial also showed a remarkable variation in the dose used in the trials. The magnesium was administered intravenously before using cisplatin, in a dose between 8 and 20 mEq (1-2.4 g), and a dose as high as 5 g IV, followed by oral replete, was also seen [13,14].

Amiloride and magnesium-sparing

Amiloride possesses magnesium-conserving properties by increasing its reabsorption in the distal nephron in addition to its well-known potassium-sparing and natriuretic effects [15,16]. A clinical trial with 18 patients with Gitelman syndrome, an autosomal recessive kidney tubule disorder, characterized by low blood levels of potassium and magnesium, demonstrated an increase of 0.18 mmol/L (0.43 mg/dL, 95% CI: 0.12-0.80, p < 0.01) and a significant reduction in urinary magnesium excretion with the use of 20 mg/day of amiloride (starting at 10 mg and titrated weekly to 15 mg/day and then to 20 mg/day) [16]. Amiloride has also been investigated for the prevention of amphotericin B (Ampho-B)-induced electrolyte disorders. Hypokalemia secondary to Ampho-B administration has been reported with an incidence as high as 75-90%. Conversely, hypomagnesemia may occur by the second week of therapy following relatively small cumulative doses of Ampho-B. Electrolyte abnormalities may persist for weeks following discontinuation of therapy. The mechanism of Ampho-B-induced hypomagnesemia is not entirely clear; however, it may involve defects in magnesium reabsorption in the distal tubule or altered body distribution of magnesium because of cell membrane effects of Ampho-B [16]. In a prospective, controlled clinical trial, ten patients received Ampho-B alone, while ten received Ampho-B and amiloride 5 mg twice daily. Hypomagnesemia requiring correction with intravenous magnesium developed in two patients receiving Ampho-B alone and in one patient receiving Ampho-B and amiloride [16-18]. In our case, we decided to use a potassium-sparing diuretic to treat the hypokalemia. However, considering that amiloride could also improve magnesium levels, we favored its use instead of spironolactone. We could not report any improvement with the drug.

SGLT2 inhibitors

A meta-analysis of 18 trials of the use of SGLT2 inhibitors with canagliflozin, empagliflozin, dapagliflozin, or ipragliflozin revealed a linear dose-dependent effect in the reduction of urinary magnesium wasting, suggesting a class effect of these drugs. However, these randomized trials were not originally designed to evaluate this effect, and the confounding factors were not controlled. A gradient effect was seen in one trial, wherein the use of canagliflozin at doses of 100 mg and 300 mg increased serum magnesium compared to placebo in patients with baseline serum magnesium <0.74 mmol/L (1.79 mg/dL), with percentages of 17.0% and 19.0% versus 3.9%, respectively [19,20]. A reduction of AKI incidence was observed with patients using SGLT2 inhibitors and nonsteroidal anti-inflammatory drugs, thiazide diuretics, anti-herpes simplex virus drugs, and loop diuretics. However, no differences were observed in patients using SGLT2 and vancomycin or cisplatin. Using SGLT2 inhibitors for cisplatin-related hypomagnesemia has been described only in case reports [19]. One more time, we tried to use one medication to treat two conditions: diabetes and hypomagnesemia. We discussed with the endocrinology team, which was planning a medication change because of poor diabetes control, and the patient started the use of dapagliflozin. This time, we were successful in having a normalization of the magnesium serum levels.

## Conclusions

Early signs of CIN commonly manifest as electrolyte disturbances, including hypomagnesemia, hypocalcemia, and hypokalemia. CIN is related to high peak plasma platinum concentrations, including doses of cisplatin over 50 mg/m^2^, previous exposure to cisplatin, preexisting kidney damage, and concomitant use of other nephrotoxic agents. Other chemotherapy agents such as ifosfamide and paclitaxel are also related to increased toxicity. Magnesium supplementation is not only a treatment for hypomagnesemia, but it is also a well-established agent in preventing CIN. Its use should be encouraged as a premedication. However, the subsequent use of magnesium for the treatment of hypomagnesemia is more challenging because of the long-lasting electrolyte imbalance, side effects of oral magnesium use, and logistical problems with the use of intravenous magnesium. A meta-analysis of SGLT2 inhibitors in diabetic patients showed a reduction of magnesium urinary wasting. Besides not being specifically designed for the evaluation of hypomagnesemia and confounding factors were not addressed, this drug class can be effective in treating this condition. Further consistent clinical studies are required before a formal recommendation can be made, and, currently, its use for hypomagnesemia is considered off-label. Its use in hypomagnesemia because of cisplatin use is restricted to case reports.
